# Impact of Physiotherapy on Shoulder Kinematics in Swimmers with Swimmer’s Shoulder Pain

**DOI:** 10.3390/s24247936

**Published:** 2024-12-12

**Authors:** Alessandra Raffini, Miriam Martini, Laura Mazzari, Alex Buoite Stella, Manuela Deodato, Luigi Murena, Agostino Accardo

**Affiliations:** 1 Department of Engineering and Architecture, University of Trieste, Via Valerio 10, 34127 Trieste, Italy; alessandra.raffini@phd.units.it (A.R.); accardo@units.it (A.A.); 2 Department of Medicine, Surgery and Health Sciences, University of Trieste, Via Pascoli 31, 34141 Trieste, Italy; miriam.martini@phd.units.it (M.M.); mdeodato@units.it (M.D.); lmurena@units.it (L.M.)

**Keywords:** inertial sensors, shoulder kinematic, swimming, physiotherapy

## Abstract

Swimmer’s shoulder is a common condition among elite swimmers, often leading to pain and reduced performance. Fatigue can exacerbate this condition by affecting shoulder strength, proprioception, and range of motion, potentially increasing the risk of overuse injuries. This preliminary study aimed to evaluate the impact of physiotherapy treatment and the effects of fatigue on shoulder kinematics using inertial and magnetic measurement units (IMUs). Five male swimmers (aged 21–27) with at least 3 years of training and suffering from swimmer’s shoulder pain participated in the study. The protocol included three sessions: dry front crawl exercises using one arm in the first and third sessions, and a fatiguing swimming exercise in the second. IMUs were used to capture 3D rotation angles, focusing on flexion/extension, abduction/adduction, and internal/external rotations during the first and third sessions. Stroke amplitude was analyzed before and after the physiotherapy treatment and fatiguing exercise. The results showed a significant increase in internal/external rotation amplitude post-fatigue before physiotherapy (*p* = 0.03), with a non-significant decrease in flexion/extension after treatment, suggesting improved shoulder stabilization. Despite these preliminary findings being based on a reduced number of participants, they indicate that physiotherapy may enhance shoulder motion control in swimmers with shoulder pain. Nevertheless, further studies with larger cohorts are needed to confirm these results.

## 1. Introduction

The shoulder joint is the most mobile in the body thanks to the interaction of bones and joints involved in this complex structure, which provide stability, balance mobility, and support. All the anatomical structures have to work in a chain to maintain the correct biomechanics of the shoulder [1,2]. The high degree of motion is due to its synovial ball-and-socket structure where the head of the humerus fits into the shallow glenoid cavity of the scapula [2]. The shoulder allows for a wide range of movements including flexion/extension, abduction/adduction, internal/external rotation, and circumduction, which is a combination of movements in all directions [3,4]. However, this wide mobility results in reduced joint stability, making the shoulder more susceptible to injury and dislocations. As a result, shoulder dysfunctions rank as the third most common musculoskeletal disorder [5] and are one of the leading causes of musculoskeletal pain and disability which cause alteration of movement, going from disorders with well-defined diagnostic criteria and pathophysiology to vaguer, undiagnosable issues with no clear definition [1,5,6].

Shoulder injuries are widespread among athletes, particularly in overhead athletes who perform shoulder movements with high intensities and extreme ranges of motion. Considering swimming, shoulder injuries are estimated to occur in 23% to 38% of swimmers within a single year [7]. Movements executed during a swim cycle involve complex and highly coordinated musculoskeletal sequences, subjecting the shoulder region to significant multidirectional stresses and forces. These demands can strain the shoulder considerably, often resulting in pain or discomfort in this anatomical area. This results in adaptive structural changes that enhance the athlete’s ability to execute the sport-specific movements more efficiently. However, these adaptations often come at the cost of normal glenohumeral joint biomechanics, increasing the risk of developing various pathological conditions over time [8,9].

Swimmer’s shoulder refers to shoulder pain experienced by swimmers, described by Kennedy and Hawkins as supraspinatus tendon impingement under the coracoacromial arch. Nowadays, many studies report that shoulder pain in swimmers should be considered multifactorial and can be caused by various conditions including subacromial impingement syndrome, overuse, muscle fatigue, scapular dyskinesis, as well as joint laxity and instability [10]. Characterizing shoulder kinematics is fundamental to the diagnosis, objective evaluation of treatment, the recognition of altered patterns, and the identification of changes [5,6,10]. In particular, in sports, the study of shoulder kinematics is also important to optimize performance and identify altered movement patterns to prevent injury [11].

Various noninvasive methods, categorized into wearable and nonwearable systems, are available for objectively analyzing shoulder kinematics. Nonwearable devices, such as ultrasound-based motion analysis systems [12], stereophotogrammetry [13], and optoelectronic systems [5], are considered the gold standard due to their reliability, accuracy, and precision [14]. However, these methodologies present several limitations in the measurement system: they are expensive, they require time-consuming data processing procedures, and their use is confined to structured laboratory settings. Wearable systems address these limitations by providing a practical and portable alternative method for evaluating kinematic parameters and facilitating the study of human behaviors over extended time periods [15,16]. Moreover, more recently, they gained significant influence in diagnostics, rehabilitation monitoring, and treating neurological and musculoskeletal disorders [14]. Wearable units (IMUs) generally include a triaxial accelerometer, a triaxial gyroscope, and a triaxial magnetometer integrated into a single device and supported by a fusion algorithm to minimize measurement errors [17]. Magnetic measurement systems enable accurate estimation of a rigid body’s 3D position and 3D orientation [14,18] and have been suggested to be useful in evaluating different joints in both the upper and lower limbs [19,20,21,22].

The upper limb can be represented as a kinematic chain of rigid segments (thorax, upper arm, forearm, and hand) connected by joints that enable relative motion between adjacent segments. Within this kinematic chain, the shoulder joint has three degrees of freedom corresponding to abduction/adduction, internal/external rotation, and flexion/extension. Thus, shoulder rotations are often described using Euler angles, aligning the anatomical degrees of freedom with roll, pitch, and yaw angles [14]. Some researchers propose methodologies to study upper limb kinematics in ambulatory settings [23], in particular, Cutti et al. [18] developed a protocol to measure scapulothoracic, humerothoracic, and elbow 3D kinematics in ambulatory settings for simple and slow movements [24,25]. Magalhaes et al. decided to define a protocol for the kinematic analysis of swimming, adapting the previous one by Cutti et al. for several reasons: the protocol was developed to be used with IMUs; is suitable for sports settings; offers a simple, quick setup during data collection; and provides a precise estimate of 3D joint angle kinematics in a clinical setting as a stereophotogrammetric system [16,26].

Since swimmer’s shoulder can be treated with different rehabilitation protocols [27,28,29], and to the best of the authors’ knowledge, kinematic differences have been scarcely used as an outcome for these treatments, in this preliminary work, we aimed to describe the effects of physiotherapy treatment and the influence of fatigue due to swimming on shoulder kinematics using IMUs. Furthermore, this is an extension of [30], in which we explore only fatigue’s influence in healthy subjects and those suffering from swimmer’s shoulder pain.

## 2. Materials and Methods

### 2.1. Participants

Male swimmers, aged between 18 and 40 years, from local swimming clubs were recruited in this study. The inclusion criteria specified a minimum of three years of swim training, an average weekly training volume of at least 4.5 h, and a primary specialization in the front crawl stroke. A team of expert physiotherapists deemed the subjects to have swimmer’s shoulder pathology, and they were excluded if the presence of primary impingement, tissue pathology, or high irritability was found. In particular, the recommendations suggested by Tovin [31] were adopted, focusing on the impairments that are associated with the onset of symptoms including glenohumeral hypermobility or instability, impaired posture, impaired rotator cuff strength, altered scapulohumeral rhythm or poor neuromuscular control, or a tight posterior capsule. Indeed, all the subjects who first reported a subjective feeling of pain in the shoulder district while swimming or immediately after received a dedicated evaluation. After the subjective assessment, which collected information about the area, symptoms description, behavior, and intensity, a physiotherapy clinical examination checked for postural abnormalities, reduced shoulder range of motion with pain, altered scapulohumeral rhythm, and impingement tests.

All participants were provided comprehensive information regarding the study’s purpose, methodology, and potential risks and benefits. The study protocol received ethical approval from the local university ethics committee (reference number 122/2022). Written informed consent was obtained from each participant before study enrolment, in accordance with ethical guidelines for human subject research.

### 2.2. Study Protocol

**Figure 1:**
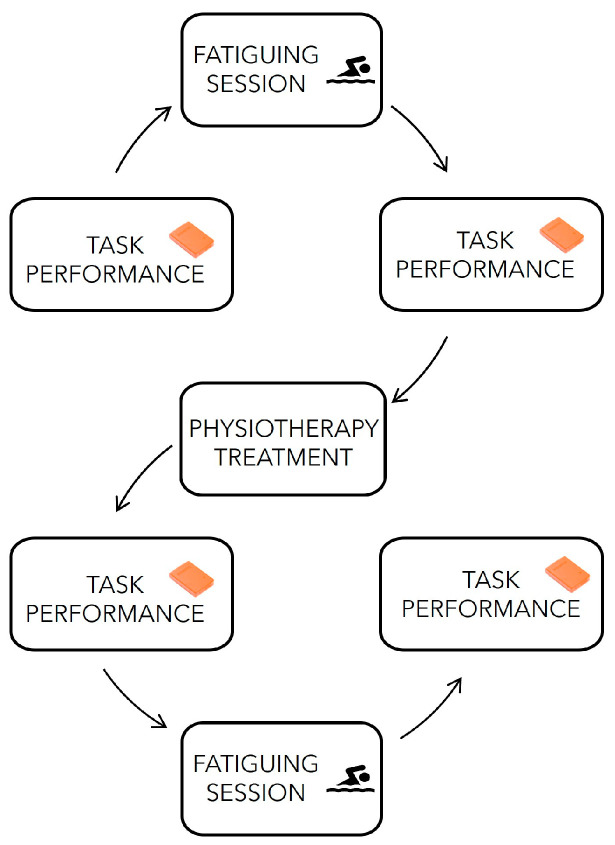
Schematic diagram representing the phases of the protocol implemented in the study.

To register the movement, we followed the same protocol as in a previous study from our group [26]; in more detail, the experimental protocol, performed in a local swimming pool, consisted of two testing tasks, separated by a standardized in-water fatiguing session (Figure 1).

In particular, during the two testing tasks, inertial sensors were applied, and participants executed a 40 s dry front crawl exercise using the pathological arm while lying prone on a physiotherapist’s bed with a metronome set at 70 beats per minute. The arm had to be over the head or along the body at each beat to perform one stroke every two beats. Moreover, the protocol provides for one calibration phase before the start of the exercise, with the subject maintaining the resting position with the arm along the body for a few seconds. The fatiguing session performed between the two testing tasks was conducted with 30 min of front crawl swimming in water at different incremental intensities following the protocol outlined in [32] and led by an expert swimming trainer. To assess that swimming intensity was the same for all the subjects, heart rate monitors and subjective ratings of perceived exertion were used to guide the exercise bouts.

Each participant was tested before and after physiotherapy treatment with ten sessions lasting about 1 h each, based on targeted physical dry exercises designed to reduce shoulder joint pain and strengthen the relevant muscles according to previous literature [31]. Manual therapy techniques were first used to address pain. Then, active exercise was promoted to correct postural deviations; improve anterior chest musculature; address hypomobility of the thoracic spine, loss of joint mobility, or excessive joint mobility; stretch the posterior capsule; and improve strength and endurance of the rotator cuff and scapular stabilizers. In particular, the endurance capacity of the pectoralis major muscle was addressed, as it was found impaired in previous research [32]. This approach aims to correct compensatory techniques that participants may have adopted in their sports movements due to pain or dysfunction related to the swimmer’s shoulder condition.

### 2.3. Acquisition and Analysis

The movements were recorded using two wireless magneto inertial sensors from the MTw Awinda Development Kit (Xsens Technology, Enschede, The Netherlands) capable of detecting 3D Euler angles. A modified version of the protocol proposed by Fantozzi et al. [11] was adopted: one sensor was placed on the flat portion of the sternum of the subject and used as a reference and the other one above the center of the humerus and posteriorly using Velcro body straps. The sensors were connected to the host system via dedicated software (MT Manager 2022) allowing data collection at a sampling frequency of 100 Hz during the first and third phases of the protocol.

The objective of this study was to characterize shoulder kinematics, with a specific focus on analyzing arm movements relative to the thorax. Given that stroke duration was synchronized with a metronome, our analysis concentrated on assessing the arm’s movement amplitude in relation to the thorax according to flexion/extension, abduction/adduction, and internal/external rotation.

We identified and analyzed individual strokes for each participant and type of rotation, excluding the initial and final strokes to avoid any potential inconsistencies. Since the temporal patterns of each rotation type were consistent across strokes, we calculated the mean amplitude by averaging it over the entire exercise period. This approach allowed us to obtain a representative measure of movement amplitude for each rotation type throughout the exercise.

To examine potential kinematic differences, we compared the mean amplitude values of the three angular rotations before and after the physiotherapy treatment, as well as before and after the fatiguing protocol. Statistical significance was assessed using the Wilcoxon signed-rank test for paired samples, allowing us to determine whether any observed changes were statistically meaningful.

All kinematic data were analyzed using a proprietary software program developed in MATLAB^®^ (R2024a).

This methodological approach aimed to provide a clearer understanding of how physiotherapy and fatigue affect shoulder movement mechanics, ultimately contributing valuable insights into rehabilitation and performance optimization.

## 3. Results

Among the eight participants who gave their initial availability to participate in the study and physiotherapy treatments, three were excluded as primary impingement was suspected and they were referred to the orthopedic surgeon for further evaluations. As such, five subjects entered the study protocol, aged between 21 and 27 years, training in swimming from 10 to 15 years, competing at the national level, and with a training volume between 10 and 12 h per week. During the subjective evaluation, participants reported suffering from “shoulder pain” from 1 to 4 years, with pain usually presenting during swimming or immediately after, usually resolving before the following day, and with a pain intensity ranging from 4 to 7. After the physiotherapy protocol, all the participants reported an improvement in overall symptoms; two of them reported a complete resolution of symptoms, while three of them reported a decrease both in the frequency of pain and its intensity (maximum 2 on 10 on a numeric rating of pain).

**Figure 2:**
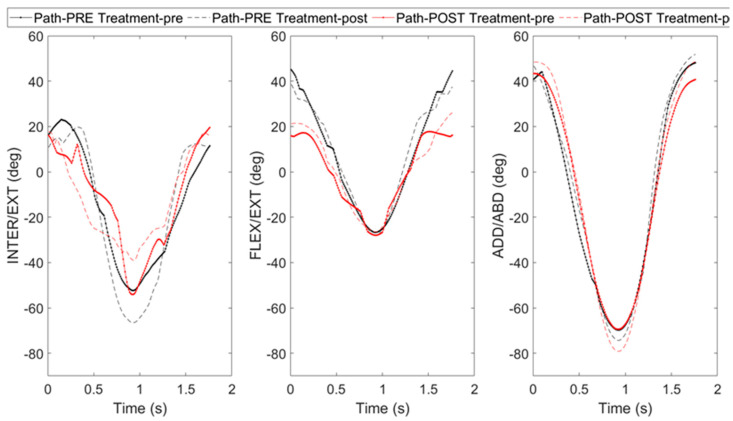
Mean amplitude trend of internal/external rotation, flexion/extension, and adduction/abduction before physiotherapy treatment (black) and pathological subjects after physiotherapy treatment (red), before (solid line), and after (dashed line) fatiguing protocol.

Figure 2 shows the mean amplitude trend of the three rotational movements in participants, both before and after physiotherapy treatment and the fatiguing protocol.

When comparing pre-treatment and post-treatment conditions before the fatiguing protocol, all three angular rotations exhibit similar patterns. However, a decrease in amplitude is noted in flexion/extension rotation. In contrast, when comparing the pre-treatment and post-treatment conditions after the fatiguing protocol, flexion/extension rotation remains relatively consistent, adduction/abduction rotation shows a slight variation, and internal/external rotation presents a pronounced difference.

To confirm the observations above, Table 1 presents the mean values (±1 SD) of stroke amplitude for each angular rotation, both before and after the fatiguing protocol and physiotherapy treatment. The values underline a large variability among the participants.

**Figure 3:**
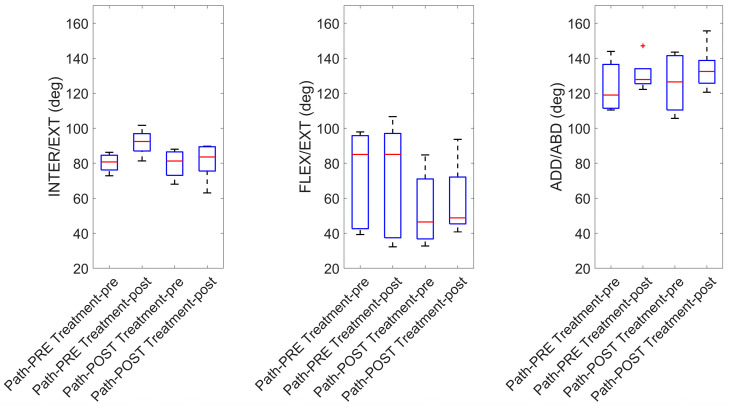
Box plots of the amplitude values for each angular rotation and condition (before and after the physiotherapy treatment and before and after the fatiguing protocol). The median values are in red with the box delimiting the 25° and 75° percentiles, and the red point indicates one outlier value.

Figure 3 presents a box plot illustrating the distribution of amplitude values for each angular rotation, highlighting the median, 25th percentile, and 75th percentile values. This visual comparison provides a clear overview of the changes in amplitude before and after the physiotherapy treatment as well as before and after the fatiguing protocol. Each box plot captures the central tendency and variability for internal/external rotation, flexion/extension, and adduction/abduction amplitudes.

The internal/external rotation amplitude presents (Table 1) an increase between before and after the fatiguing exercise in the pre-treatment condition from 80 degrees to 92 degrees, while flexion/extension shows a large decrease from 71 degrees to 59 degrees in amplitude after the fatiguing protocol between before and after the treatment. Additionally, the adduction/abduction amplitude reveals an increase between before and after the fatiguing protocol both pre- and post-physiotherapy treatment.

Due to the small sample size and high variability in participant responses, statistically significant changes were observed only for the internal/external rotation amplitude in the pre-treatment condition, which showed a significant increase post-fatigue (*p* = 0.03). A trend toward statistical significance was also observed for internal/external rotation amplitude, with *p*-values of 0.06 approaching significance when comparing the pre- and post-treatment measurements after the fatiguing protocol. Moreover, flexion/extension amplitude also exhibited a *p*-value near significance (*p* = 0.06) in the pre- and post-treatment comparison conducted before the fatigue exercise.

## 4. Discussion

This preliminary study investigates the effects of a physiotherapy treatment and the impact of fatigue from swimming on shoulder kinematics, utilizing two IMUs placed on the sternum and humerus. The research focuses on individuals experiencing swimmer’s shoulder pain, a prevalent and debilitating condition among elite swimmers. Swimmer’s shoulder not only affects performance but also poses a significant risk to long-term shoulder health, highlighting the importance of developing effective diagnostic and rehabilitation strategies. These preliminary findings demonstrate that magneto-inertial sensors provide precise quantification of shoulder rotation angles and allow for detailed measurement of stroke amplitude during dry-land simulations of the front crawl exercise. This technology offers valuable insights into kinematic patterns, enabling a better understanding of how fatigue and physiotherapy interventions influence shoulder mechanics.

Swimming is a sport that requires high flexibility in terms of joint hypermobility defined as the ability to move the joints beyond the typical range of motion [31]. From an anatomical perspective, this hypermobility is linked to impaired collagen, resulting in laxity of the connective tissue matrix. Such laxity compromises the stability of the joint capsule and increases the extensibility of ligaments, tendons, and skin, requiring a more intense stabilizing muscular action. However, while joint hypermobility may confer certain advantages, such as enhanced flexibility and range of motion, it could lead to significant risks. Athletes with hypermobile joints are more susceptible to sport-related injuries due to potential deficits in muscle strength, greater muscular fatigue, and compromised joint stability [31]. Understanding these biomechanical dynamics is crucial for tailoring physiotherapy interventions aimed at minimizing injury risks while optimizing performance in elite swimmers [33,34].

In particular, in these athletes, injuries are frequently attributed to specific adaptations in stabilizing structures, resulting from the repetitive and intense demands of the sport. Initially, these adaptations seem to enhance the efficiency of the performance; however, over time, they can lead to alterations in shoulder biomechanics. It is important to note that such adaptations may appear not only after several years of practice but even after a single sports season [35]. Walker et al. [36] report that adolescent swimmers having an internal/external rotation of more than 100° could develop shoulder injuries, supporting the hypothesis that an optimal range of flexibility is necessary to swim without risking shoulder injury, and Behnam Liaghat et al. [37] found that young competitive swimmers with generalized hypermobility show both strength and fatigue deficits in medial rotation.

The results of this study reveal a statistically significant increase (*p* = 0.03) in internal/external rotation amplitude in the pre-treatment condition when comparing values before and after the fatiguing exercise. Additionally, a reduction in amplitude was observed after the fatigue protocol when comparing the pre-treatment to post-treatment conditions. The findings about internal/external rotation are particularly intriguing, as these movements are markedly involved in front crawl swimming and swimmer’s shoulder [31]. The increase in internal/external rotation amplitude observed immediately after the fatiguing exercise may be attributed to the activation of muscle memory, developed through repetitive in-water training [38]. This muscle memory likely enables swimmers to maintain or even enhance movement efficiency during fatigue states. However, while such adaptations may initially aid performance, they could also reflect compensatory strategies that place additional strain on the shoulder joint, potentially increasing the risk of injury over time. Indeed, it has been previously suggested that a similar fatiguing task during swimming might result in altered muscular responses of the latissimus dorsi and pectoralis major muscles [32] that could, therefore, influence internal/external rotation amplitude; during the physiotherapy treatment, specific attention was directed to these muscles, trying to improve not only their strength and endurance capacity but also their pattern of activation. As such, it might be speculated that the reported differences might depend on the role of these muscles, how fatigue affects their activation, and how the treatment has improved their function and control.

The flexion/extension amplitude shows a large decrease after the fatiguing protocol both before and after the treatment, with the post-treatment amplitude values being notably lower. Swimmers often exaggerate scapular protraction as a technique to achieve a wider and more powerful stroke and improve performance. While this adaptation may provide short-term benefits in the water, it places excessive strain on the shoulder complex, leading to pain and injuries. The primary goal of physiotherapy treatment in such cases is to target these biomechanical challenges by strengthening the stabilizing muscles of the shoulder, helping to restore and maintain a healthy and functional range of motion. This approach not only helps to alleviate pain but also enhances movement efficiency, promoting proper biomechanics and significantly reducing the risk of future injuries.

The adduction/abduction amplitude values remain similar when comparing the pre-treatment and post-treatment measurements, with only a slight increase observed after the fatiguing protocol. Similar to the trends observed in internal/external rotation, this increase in amplitude could be attributed to muscle memory developed through the repetitive in-water movements characteristic of swimming.

The use of wearable sensors to assess shoulder kinematics has been proposed as a promising solution to provide the clinician and physiotherapist with reliable and quantitative data regarding the functional evaluation of the shoulder to support a better diagnosis and support injury prevention programs [39], representing an objective marker of rehabilitation efficacy as well [14]. Indeed, by providing such quantitative measures, it might be possible to better define the pathophysiological mechanisms underlying such a complex complaint that might manifest in a wide range of conditions and populations, from the elderly to the occupation setting and to elite athletes, with a great variety of symptoms. Among them, surface electromyography, force sensors, inertial measurement units (IMU), accelerometers, fiber optic sensors, and strain sensors have been widely applied. With such techniques and their application within the “big data” context, machine learning might be implemented to further improve home rehabilitation by recognizing the quality of performed physical exercises and possibly preventing disorders in patients’ movement [40]. As such, these devices might be useful to tailor the most optimal treatment to a clinical condition characterized by a heterogeneous clinical and symptomatic manifestation.

In swimming, the use of wearables has been implemented to provide a wide range of information, including athletic performance and training load, or swimming technique kinematics [41,42,43]; however, to the best of the authors’ knowledge, these are the first preliminary findings about the use of wearables, as IMUs, to evaluate the efficacy of physiotherapy in the treatment of “swimmer shoulder” pain.

In conclusion, the use of magneto inertial sensors allows for the identification of the specific movement components affected by the pathology, which could help provide a more targeted treatment. Due to the restricted sample size and the large variability, we found only one significant difference before and after the fatiguing exercise, indicating that fatigue impacts shoulder movement patterns. Additionally, a few differences approached statistical significance when comparing the pre- and post-physiotherapy treatment results. Among the other limitations, the absence of a healthy control sample might have limited the interpretation of the current findings, as well as a comparison with the contralateral limb. Nevertheless, it should be reported that the proposed task is in line with some previous works addressing the “swimmer shoulder”. Moreover, to the best of the authors’ knowledge, this is the first study addressing the effect of a fatiguing task on these parameters and in this specific population, the latter being characterized by a selected form of shoulder pain.

Further investigation with larger sample sizes and more diverse fatigue protocols is needed to clarify the biomechanical implications of these subtle changes and to determine how physiotherapy interventions might optimize this aspect of shoulder kinematics in swimmers. Moreover, considering both sexes could determine if differences are present between male and female swimmers, as well as between different swimming techniques.

## 5. Conclusions

This pilot study aimed to assess the impact of physiotherapy on subjects experiencing swimmer’s shoulder pain, focusing specifically on changes in shoulder kinematics. The study’s methodology allowed for the assessment of movement repeatability and provided quantitative data on shoulder rotation angles across different movement planes. Despite the small sample size, key findings included a significant difference in internal/external rotation amplitude after the fatiguing exercise before physiotherapy treatment. Additionally, a reduction of approximately ten degrees in flexion/extension rotation amplitude was observed post-physiotherapy, suggesting improved shoulder stability and enhanced motor control.

These preliminary results highlight the potential of physiotherapy in promoting shoulder stabilization and modifying movement patterns that may contribute to injury prevention. However, increasing the sample size is crucial to substantiate these findings and gain deeper insights into the kinematic adaptations associated with physiotherapy, aiming to recruit and test at least 15 subjects to better highlight the role of internal/external rotation. A larger cohort would also allow for a more comprehensive analysis of how these adaptations might mitigate the risk of shoulder injuries in swimmers and other overhead athletes. Future research with expanded participant numbers is essential to confirm these effects and further explore physiotherapy’s role in optimizing shoulder function and preventing sports-related injuries.

## Figures and Tables

**Table 1:** Mean values (±1 SD) of stroke amplitude [°] before and after physiotherapy and fatiguing exercise for each of the three rotations.

		Internal/External [°]	Flexion/Extension [°]	Abduction/Adduction [°]
Before Treatment	Before fatigue	80 ± 5	72 ± 28	124 ± 15
	After fatigue	92 ± 8	71 ± 34	131 ± 10
After Treatment	Before fatigue	80 ± 8	54 ± 22	126 ± 17
	After fatigue	81 ± 11	59 ± 21	134 ± 13

## Data Availability

Raw data can be requested to the corresponding author.
